# Propensity Score Matching Analysis of Changes in Alpha-Fetoprotein Levels after Combined Radiotherapy and Transarterial Chemoembolization for Hepatocellular Carcinoma with Portal Vein Tumor Thrombus

**DOI:** 10.1371/journal.pone.0135298

**Published:** 2015-08-07

**Authors:** Yuri Jeong, Sang Min Yoon, Seungbong Han, Ju Hyun Shim, Kang Mo Kim, Young-Suk Lim, Han Chu Lee, So Yeon Kim, Jin-hong Park, Sang-wook Lee, Seung Do Ahn, Eun Kyung Choi, Jong Hoon Kim

**Affiliations:** 1 Department of Radiation Oncology, Asan Medical Center, University of Ulsan College of Medicine, Seoul, Korea; 2 Department of Biostatistics, Asan Medical Center, University of Ulsan College of Medicine, Seoul, Korea; 3 Department of Gastroenterology, Asan Medical Center, University of Ulsan College of Medicine, Seoul, Korea; 4 Department of Radiology, Asan Medical Center, University of Ulsan College of Medicine, Seoul, Korea; 5 Asan Liver Center, Asan Medical Center, University of Ulsan College of Medicine, Seoul, Korea; University of Modena & Reggio Emilia, ITALY

## Abstract

**Background and Aim:**

To investigate the value of changes in alpha-fetoprotein (AFP) levels for the prediction of radiologic response and survival outcomes in hepatocellular carcinoma (HCC) patients with portal vein tumor thrombus (PVTT) who received combined treatment of 3-dimensional conformal radiotherapy (3D-CRT) and transarterial chemoembolization (TACE).

**Methods:**

A database of 154 HCC patients with PVTT and elevated AFP levels (>20 ng/mL) treated with 3D-CRT and TACE as an initial treatment between August 2002 and August 2008 was retrospectively reviewed. AFP levels were determined 1 month after radiotherapy, and AFP response was defined as an AFP level reduction of >20% from the initial level. Radiologic response, overall survival (OS), and progression-free survival (PFS) rates were compared between AFP responders and non-responders. Propensity-score based matching analysis was performed to minimize the effect of potential confounding bias.

**Results:**

The median follow-up period was 11.1 months (range, 3.1–82.7 months). In the propensity-score matching cohort (92 pairs), a best radiologic response of CR or PR occurred in more AFP responders than AFP non-responders (41.3% vs. 10.9%, p < 0.001). OS and PFS were also longer in AFP responders than in non-responders (median OS 13.2 months vs. 5.6 months, p < 0.001; median PFS 8.7 months vs. 3.5 months, p < 0.001).

**Conclusions:**

AFP response is a significant predictive factor for radiologic response. Furthermore, AFP response is significant for OS and PFS outcomes. AFP evaluation after combined radiotherapy and TACE appears to be a useful predictor of clinical outcomes in HCC patients with PVTT.

## Introduction

Despite surveillance programs for hepatocellular carcinoma (HCC) in high-risk populations, most patients are diagnosed with advanced HCC with vascular invasions, and are therefore not eligible for curative treatment. The prognosis of advanced HCC with portal vein tumor thrombus (PVTT) is extremely poor [1]. Because PVTT itself can cause intrahepatic dissemination or extrahepatic metastasis and deteriorate liver function, it is a major obstacle to determining effective treatment options. Although the Barcelona Clinic Liver Cancer (BCLC) staging and treatment strategy indicates that sorafenib is the only recommended treatment for advanced stage patients [2], the survival gain is modest and response rates are relatively low [3]. Partly because of these somewhat disappointing results, other various modalities such as transarterial chemoembolization (TACE), radioembolization, hepatic intra-arterial chemotherapy, external beam radiotherapy, and surgical resection in selected cases have been attempted before and after the use of sorafenib.

To reduce or stabilize PVTT and maintain portal blood flow, 3-dimensional conformal radiotherapy (3D-CRT) with or without TACE has shown promising clinical outcomes and safety in several studies [4–8]. Despite the effectiveness of this combined treatment, most patients experience recurrences during the follow-up periods. Moreover, accurate radiologic assessment of the treated HCC is difficult in cases of infiltrative primary tumors or in the presence of underlying liver cirrhosis. Therefore, additional tools to radiological evaluation are needed to assess the prognosis of patients with advanced HCC.

The tumor marker alpha-fetoprotein (AFP) is secreted in 39–65% of HCC patients, and has been used as a diagnostic tool [9,10]. Based on the hypothesis that AFP levels reflect the tumor activity and burden, this marker has been frequently measured during the treatment. In several studies, AFP response has been reported as a meaningful predictive factor for radiologic response, recurrence, and survival in early and advanced HCC cases [11–16]. Although 3D-CRT with or without TACE has been used to treat advanced HCC patients with PVTT, the predictive value of AFP levels after this combined treatment has not been assessed previously. Therefore, we here investigated changes in AFP levels for the prediction of radiologic response and survival outcomes in advanced HCC patients with PVTT who received combined treatment of 3D-CRT and TACE.

## Methods

### Ethics statement

This study was approved by the Institutional Review Board of the Asan Medical Center, and written informed consents were obtained from all patients.

### Patients

Among the 412 HCC patients who were treated with TACE and 3D-CRT for PVTT between August 2002 and August 2008 in our previous report [7], 229 patients underwent combined 3D-CRT and TACE as an initial treatment after diagnosis of advanced HCC. Of these, 30 patients with an AFP level of ≤20 ng/mL and 45 patients who had no AFP evaluation after treatment were excluded from the present analysis. The remaining 154 patients were retrospectively reviewed.

### Treatment

The combination treatment procedure has been described in detail in our previous report [7]. For the TACE procedure, a mixture of 2–10 mL of iodized oil (Lipiodol; Laboratoire Andrè Guerbet, Aulnay-sous-Bois, France) and 1 mg/kg cisplatin (Cisplatin; Dong-A Pharm. Co. Ltd, Seoul, Korea) was infused with or without embolization using gelatin sponge cubes (Gelfoam; Upjohn, Kalamazoo, MI). Radiotherapy was planned after the identification of PVTT at initial presentation and was started 2–3 weeks after TACE. All patients underwent simulation with a CT scanner (LightSpeed RT 16, GE Healthcare, Waukesha, WI). Radiotherapy was performed with 6- or 15-MV X-rays from a linear accelerator (Varian, Palo Alto, CA). The fraction size was 2 to 5 Gy, and the total dose was determined by the volume of normal liver, liver function, and the maximum dose to the stomach or duodenum [7].

### Evaluation

Serum AFP levels were measured by chemiluminescent microparticle immunoassay (ARCHITECT i2000SR; Abbott, Chicago, IL). AFP response was defined as an AFP reduction of >20% (compare to the initial level) at 1 month after completion of radiotherapy. Radiologic response evaluation was performed by multiphase dynamic CT scans according to the modified Response Evaluation Criteria in Solid Tumors (mRECIST) criteria [17].

### Statistics

Patient characteristics were compared between AFP responders and AFP non-responders using the student t, χ², and Fisher’s exact test. Overall survival (OS) and progression-free survival (PFS) rates were calculated from the date of start of treatment to the date of death or last follow-up, and to the date of progression of HCC and/or PVTT, distant metastasis, death or last follow-up, respectively, according to the Kaplan-Meier method and compared between the two groups by the log-rank test. A Cox proportional hazards model was used to generate the univariate and multivariate models describing the association of variables with OS and PFS. Backward elimination Cox’s regression was used to select the principal risk factors in the multivariate model. Variables with p values ≤ 0.2 by univariate analysis were chosen for multivariate analysis. To reduce potential confounding effects in this retrospective study, propensity-score based matching analysis was performed, which included all possible variables. We performed caliper matching on the PS (nearest available matching). Pairs (AFP responders and AFP non-responders) on the PS logit were matched to within a range of 0.2 multiplied by the standard deviations [18]. The balance of covariates was measured by their standardized differences. A difference of >20% of the absolute value was considered significantly imbalanced. All statistical tests were two-sided and performed at the 5% level of significance using SPSS version 18.0 (SPSS Inc., Chicago, IL) and R software version 2.13 (R Foundation for Statistical Computing, Vienna, Austria; www.r-project.org).

## Results

Patient characteristics are summarized in Table 1. The initial AFP level was ≤400 ng/mL in 52 (33.8%) patients. The median total radiation dose was 41.7 Gy (equivalent dose in 2 Gy fractions (EQD2), α/β = 10) (range, 26–62.5 Gy). Of the 154 patients, 99 (64.3%) were AFP responders and 55 (35.7%) were AFP non-responders. Liver function was significantly better (Child-Pugh A 64.6% vs. 43.6%, p = 0.012) and tumor size was smaller (median 10.3 cm vs. 12.0 cm, p = 0.027) in AFP responders than in AFP non-responders. There were more patients with hepatitis B virus (HBV) infection among AFP non-responders than among AFP responders. Otherwise, no other significant differences in patient characteristics were found between the two groups. Propensity-score matching generated 92 matched pairs of AFP responders and AFP non-responders. In this matched cohort, there were no significant differences in baseline characteristics between AFP responders and AFP non-responders (Table 1).

**Table 1:** Patient characteristics.

		Entire patients			Propensity score matched patients (92 pairs)		
		AFP responder	AFP non- responder	*p-value* ^*a*^	AFP responder	AFP non- responder	*p-value* ^*b*^
		(n = 99)	(n = 55)		(n = 46)	(n = 46)	
Characteristics		No. (%)	No. (%)		No. (%)	No. (%)	
Age (years)				0.074			0.570
	Median (range)	53 (30–79)	48 (36–73)		52 (30–79)	51 (36–73)	
Gender				0.583			0.758
	Male/Female	86/13	46/9		41/5	39/7	
Child-Pugh class				0.012			0.677
	A	64 (64.6)	24 (43.6)		21 (45.7)	24 (52.2)	
	B	35 (35.4)	31 (56.4)		25 (54.3)	22 (47.8)	
ECOG performance status				0.763			0.814
	0	18 (18.2)	11 (20.0)		7 (15.2)	10 (21.7)	
	1	70 (70.7)	36 (65.5)		32 (69.6)	30 (65.2)	
	2	11 (11.1)	8 (14.5)		7 (15.2)	6 (13.0)	
Initial AFP (ng/mL)				0.879			1.000
	≤ 400	33 (33.3)	19 (34.5)		13 (28.3)	14 (30.4)	
	> 400	66 (66.7)	36 (65.5)		33 (71.7)	32 (69.6)	
Viral etiology				0.041			1.000
	HBsAg (+)	85 (85.9)	53 (96.4)		44 (95.7)	44 (95.7)	
	HBsAg (-)	14 (14.1)	2 (3.6)		2 (4.3)	2 (4.3)	
Tumor size (cm)				0.027			0.993
	Median (range)	10.3 (2.5–21.0)	12.0 (3.0–18.0)		11.1 (3.0–21.0)	11.1 (3.0–18.0)	
Sites of PVTT				0.068			1.000
	Main or bilateral	46 (46.5)	34 (61.8)		25 (54.3)	26 (56.5)	
	Unilateral	53 (53.5)	21 (38.2)		21 (45.7)	20 (43.5)	
Modified UICC stage				0.298			0.875
	III	15 (15.2)	5 (9.1)		4 (8.7)	5 (10.9)	
	IVA	76 (76.8)	42 (76.4)		37 (80.4)	35 (76.1)	
	IVB	8 (8.1)	8 (14.5)		5 (10.9)	6 (13.0)	

*Abbreviations*: AFP = Alpha-fetoprotein; ECOG = Eastern Cooperative Oncology Group; HBsAg = hepatitis B surface antigen; UICC = International Union Against Cancer; PVTT = portal vein tumor thrombus. *p-value* ^*a*^, student t, χ², and Fisher exact test; *p-value* ^*b*^, weighted student t, χ², and Fisher exact test using propensity score matching (92 matched pairs).

The best radiologic response was complete response (CR) or partial response (PR) in 56 (36.4%) patients and stable disease (SD) or progressive disease (PD) in 98 (63.6%) patients. The median interval from the start of treatment to the best radiologic response was 2.8 months (range, 1.4–8.8 months). The best radiologic response of CR or PR was higher in AFP responders than in AFP non-responders (50.5% vs. 10.9%, p < 0.001). In the propensity-score matching cohort, more patients had a best radiologic response of ≥PR and SD among AFP responders than among AFP non-responders (CR or PR, 41.3% vs. 10.9%; SD, 34.8% vs. 17.4%, p < 0.001). More patients with best radiologic response of PD existed in AFP non-responders than in AFP responders (71.7% vs. 23.9%, p < 0.001) (Table 2).

**Table 2:** Best radiologic responses.

	Entire patients (n = 154)				Propensity score matched patients (92 pairs)			
		AFP responder	AFP non-responder	*p-value*		AFP responder	AFP non-responder	*p-value*
Response		No. (%)	No. (%)			No. (%)	No. (%)	
Complete response	8 (5.2)	8 (8.1)	0 (0)	< 0.001	1 (1.1)	1 (2.2)	0 (0)	< 0.001
Partial response	48 (31.2)	42 (42.4)	6 (10.9)		23 (25.0)	18 (39.1)	5 (10.9)	
Stable disease	36 (23.4)	26 (26.3)	10 (18.2)		24 (26.1)	16 (34.8)	8 (17.4)	
Progressive disease	62 (40.3)	23 (23.2)	39 (70.9)		44 (47.8)	11 (23.9)	33 (71.7)	

**Fig 1:**
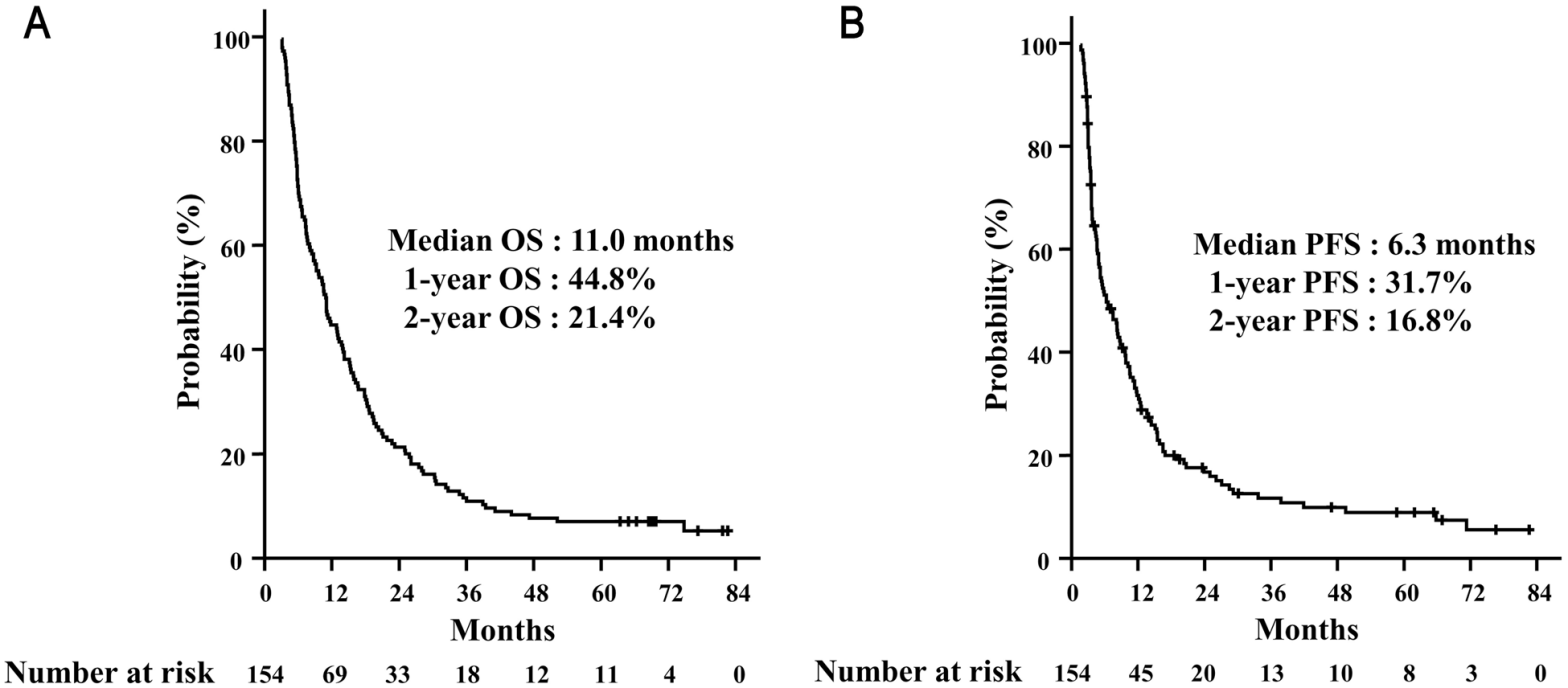
Survival outcomes. Overall survival rates (A) and progression-free survival rates (B) in all patients.

**Fig 2:**
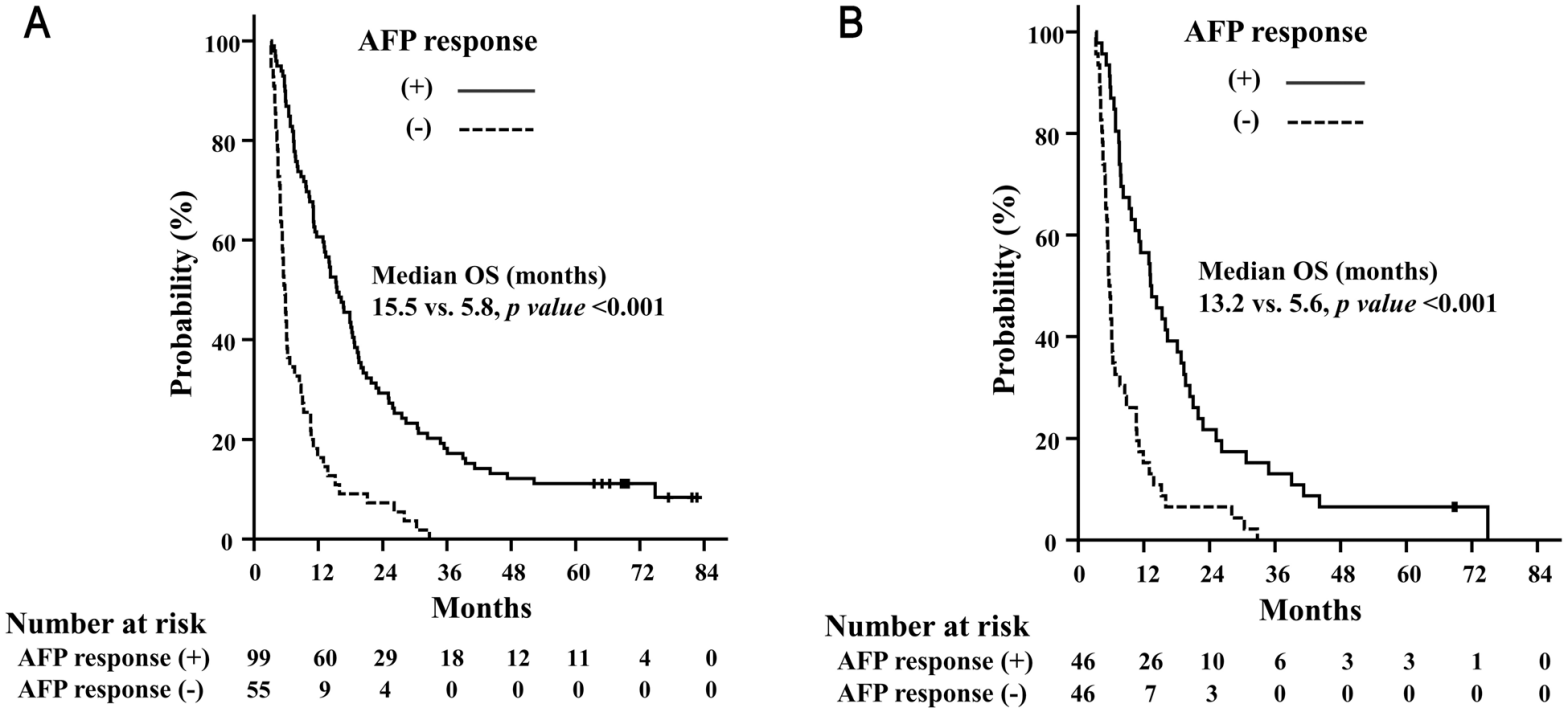
Overall survival rates. Overall survival (OS) rates depending on the AFP response in all patients (A) and in the propensity score-matching cohort (B).

**Fig 3:**
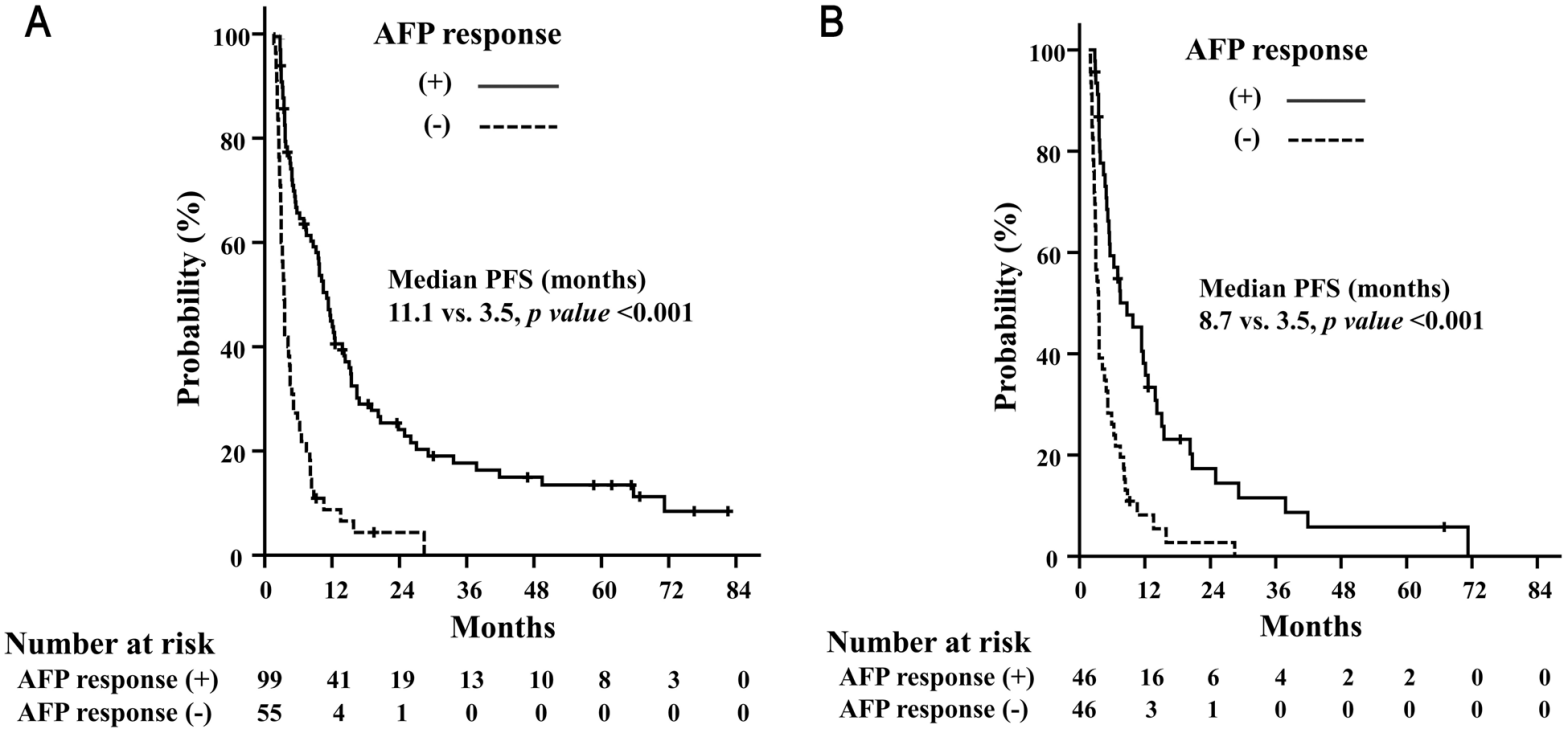
Progression-free survival rates. Progression-free survival (PFS) rates depending on the AFP response in all patients (A) and in the propensity score-matching cohort (B).

The median follow-up period was 11.1 months (range, 3.1–82.7 months). The median OS was 11.0 months and 1- and 2-year OS was 44.8% and 21.4%, respectively (Fig 1). The median OS was longer in AFP responders than in AFP non-responders (Fig 2A). PFS was also longer in AFP responders than in AFP non-responders (Fig 3A). In the propensity-score matching cohort (92 pairs), AFP responders still showed superior OS (AFP responders vs. AFP non-responders, median 13.2 months vs. 5.6 months, p < 0.001) and PFS (AFP responders vs. AFP non-responders, median 8.7 months vs. 3.5 months, p < 0.001) (Figs 2B and 3B).

On multivariate analysis, AFP response and best radiologic response were significant predictive factors for OS (AFP responder, HR = 0.312, p < 0.001; best radiologic response of CR or PR, HR = 0.213, p < 0.001) and PFS (AFP responder, HR = 0.416, p = 0.001; best radiologic response of CR or PR, HR = 0.231, p < 0.001) (Table 3). In the propensity-score matching analysis which included age, gender, Child-Pugh class, performance status, initial AFP level (≤400 ng/mL vs. >400 ng/mL), viral etiology, tumor size, sites of PVTT, and stage, AFP response remained a significant prognostic factor for OS (AFP responder, HR = 0.264, p < 0.001) and PFS (AFP responder, HR = 0.307, p < 0.001) (Table 4).

**Table 3:** Multivariate analysis for progression-free survival (PFS) and overall survival (OS) rates in the propensity score-matching cohort.

	PFS		OS	
Variables	HR (95% CI)	*P value*	HR (95% CI)	*P value*
Gender (male)	1.985 (1.030–3.828)	0.041		
Child-Pugh class (A)	1.367 (0.831–2.246)	0.218	1.597 (1.014–2.514)	0.043
ECOG performance status (0–1)			1.422 (0.769–2.629)	0.261
Initial AFP (≤ 400 ng/mL)	1.977 (1.143–3.420)	0.015	2.256 (1.320–3.856)	0.003
Tumor size (cm)	1.021 (0.955–1.091)	0.543	1.016 (0.951–1.085)	0.639
Modified UICC stage		0.014		0.173
IVA (III)	0.811 (0.344–1.912)	0.633	0.821 (0.353–1.910)	0.646
IVB (III)	2.283 (0.792–6.585)	0.127	1.560 (0.562–4.333)	0.393
AFP response (-)	0.416 (0.253–0.682)	0.001	0.312 (0.189–0.516)	< 0.001
Best radiologic response (-)	0.231 (0.115–0.466)	< 0.001	0.213 (0.114–0.400)	< 0.001

*Abbreviations*: PFS = progression-free survival; OS = overall survival; HR = hazard ratio; CI = confidence index; ECOG = Eastern Cooperative Oncology Group; AFP = Alpha-fetoprotein; UICC = International Union Against Cancer; Variables with p values ≤ 0.2 by univariate analysis were chosen for multivariate analysis.

**Table 4:** Hazard ratio (HR) for clinical outcomes in the AFP responder group compared with the AFP non-responder group.

	Unadjusted		Multivariable adjusted^a^		Adjusted by propensity matching^b^	
Outcomes	HR (95% CI)	*p*	HR (95% CI)	*p*	HR (95% CI)	*p*
OS	0.313 (0.219–0.447)	< 0.001	0.435 (0.291–0.650)	< 0.001	0.264 (0.175–0.400)	< 0.001
PFS	0.291 (0.200–0.423)	< 0.001	0.508 (0.341–0.757)	0.001	0.307 (0.206–0.457)	< 0.001

Reference = AFP non-responder group

*Abbreviations*: OS = overall survival; PFS = progression-free survival; HR = hazard ratio; CI = confidence index; AFP = Alpha-fetoprotein;

^a^Adjusted age, gender, Child-Pugh class, performance status, Initial AFP (≤ 400 ng/mL vs. >), viral etiology, tumor size, sites of portal vein tumor thrombus, stage, and best radiologic response;

^b^all the possible variables (age, gender, Child-Pugh class, performance status, Initial AFP (≤ 400 ng/mL vs. >), viral etiology, tumor size, sites of portal vein tumor thrombus, stage) were included for the propensity score matching.

## Discussion

AFP response has not only been reported as a meaningful predictive factor for recurrence and survival in early stage HCC patients who receive curative treatments [13,15,16] but also has shown the prognostic value for radiologic response and survival rates in advanced HCC after palliative treatments [11,12,14,19–21]. Regarding patients with PVTT, few studies to date have analyzed the prognostic value of AFP response after treatment. In a study by Riaz et al., 55.8% of patients showed an AFP response after TACE or radioembolization, and AFP responders had higher radiologic response rates and significantly better survival rates than AFP non-responders [14]. In two studies which analyzed prognostic value of AFP response after concurrent chemoradiotherapy, 68% to 78% of patients achieved an AFP response [12,19]. The median survival duration in those studies were similar to that of our present study, with values ranging from 13.3 to 17.6 months in AFP responders, which is significantly better than the values reported for AFP non-responders. Recently, 3D-CRT was used with or without TACE to reduce PVTT and to maintain portal blood flow [4–6,8]. However, no studies have yet analyzed the prognostic value of the AFP response after a combined 3D-CRT and TACE in patients with PVTT. In our present analyses, AFP responders showed better survival and higher radiologic response rates than AFP non-responders in all patients. In addition, the AFP responders showed a significantly better median survival of 13.2 months and higher radiologic response rates than AFP non-responders in the propensity-score matching cohort after combined 3D-CRT and TACE.

Based on the criteria used in previous sorafenib studies, we defined an AFP response as a reduction of >20% from the initial level. To improve the specificity and positive predictive value, other studies have alternatively defined the AFP response as a reduction of >50% compared to the initial level [14,15,20]. However, the proportion of AFP responders in those studies were similar to that of the present study, which may be due to similar evaluation times of AFP response used among studies. In earlier studies that define AFP response as a >50% reduction, the timing of the AFP responses were later than that in studies that define this response as a 20% reduction [14,20]. In HCC patients with PVTT, most patients have a poor prognosis and experience tumor progression during subsequent treatment. Hence, an early AFP evaluation may be more useful to determine additional treatments if the ratio of responders to non-responders is similar.

The radiologic response rate in our current study subjects was 36.4% according to mRECIST criteria. The radiologic response was found to be the strongest prognostic factor for OS and PFS, and significantly more of our AFP responder subjects achieved a radiologic response of CR or PR than the non-responders. The median time to best radiologic response from the start of treatment was 2.8 months (range, 1.4–8.8 months). This range suggests varied and unpredictable durations when compared to AFP responses that were usually evaluated at a constant timing. Riaz et al. also reported the time to best radiologic response and AFP response [14]. According to the WHO criteria, these authors found that a radiologic response was achieved in 53% of AFP responders and in 24% of AFP non-responders. The median time to best radiologic response was 5.6 months (range, 3.9–8.5 months) and the median time to AFP response was 3.3 months (range, 2.5–4.4 months). The authors suggested that an AFP response may be able to predict treatment response before a radiologic response evaluation. Other studies have evaluated radiologic response, and similar to our present study found higher radiologic response rates among AFP responders [11,19]. As we noted above, the timing of radiologic response varies between studies. Considering the prognostic value of the radiologic response rate for survival outcomes, the evaluation of the AFP response may be a useful tool to predict subsequent radiologic response in advanced HCC patients.

This study had several limitations of note. First, AFP elevation may be associated with chronic liver diseases such as viral hepatitis and liver cirrhosis as well as HCC. In patients with chronic liver disease, elevated AFP levels may reflect hepatic regeneration that occurs after parenchymal damage [22]. In our present study, 89.6% of patients had HBV infection, but the activity of hepatitis and use of antiviral agents were not considered in our analysis. Second, the prognostic value of the AFP response is limited to patients with elevated AFP levels. In our present study cohort, 30 patients were excluded from the analysis due to an initial AFP level of ≤20 ng/mL among 229 patients. Third, as mentioned above, most patients had HBV infection, which is a higher rate than observed in western counties. Because the effect of viral etiology on the prognostic value of AFP is uncertain, careful interpretation is needed. Lastly, because of the retrospective nature of our present study, baseline characteristics were not evenly distributed between the response groups at initial analysis. To address this problem, additional propensity-score matching analysis was performed and the prognostic value of the AFP response was also confirmed in this additional analysis.

Despite the abovementioned limitations, our present study is one of few investigations to evaluate the prognostic value of the AFP response after combined 3D-CRT and TACE as an initial treatment in HCC patients with PVTT. In advanced HCC patients with PVTT, the assessment of radiologic response after treatment can be difficult due to diffusely infiltrative tumors, underlying liver cirrhosis, or parenchymal changes after previous treatments. In addition, most patients with advanced HCC experience recurrences or progressions during the follow-up periods, Hence, the AFP response may be a useful tool that may predict the radiologic response and thus be used for the early prediction of clinical outcomes in HCC cases. This would assist clinicians to determine the most appropriate additional treatments.

In summary, the AFP response is a significant predictive factor for the radiologic response. Furthermore, the AFP response is statistically significantly associated with OS and PFS. AFP evaluation after combined radiotherapy and TACE thus appears to be a useful tool to predict clinical outcomes in HCC patients with PVTT.

## Supporting Information

S1 Table Raw data excel file for Tables 1 and 2, Figs 1A, 1B, 2A and 3A. This raw data excel file shows patients characteristics (age, gender, Child-Pugh class, ECOG performance status, initial AFP level, viral etiology, tumor size, site of PVTT, and modified UICC stage), treatment outcomes (AFP response and best radiologic response), and oncologic outcomes (PFS and OS) for entire patients (n = 154).
(XLS)

S2 Table Raw data excel file for Tables 1, 2, 3 and 4, Figs 2B and 3B. This raw data excel file shows patients characteristics (age, gender, Child-Pugh class, ECOG performance status, initial AFP level, viral etiology, tumor size, site of PVTT, and modified UICC stage), treatment outcomes (AFP response and best radiologic response), and oncologic outcomes (PFS and OS) for propensity score matched patients (92 pairs).
(XLS)
